# Skeletal muscle depletion during chemotherapy has a large impact on physical function in elderly Japanese patients with advanced non–small-cell lung cancer

**DOI:** 10.1186/s12885-017-3562-4

**Published:** 2017-08-25

**Authors:** Tateaki Naito, Taro Okayama, Takashi Aoyama, Takuya Ohashi, Yoshiyuki Masuda, Madoka Kimura, Hitomi Shiozaki, Haruyasu Murakami, Hirotsugu Kenmotsu, Tetsuhiko Taira, Akira Ono, Kazushige Wakuda, Hisao Imai, Takuya Oyakawa, Takeshi Ishii, Shota Omori, Kazuhisa Nakashima, Masahiro Endo, Katsuhiro Omae, Keita Mori, Nobuyuki Yamamoto, Akira Tanuma, Toshiaki Takahashi

**Affiliations:** 1 Division of Thoracic Oncology, Shizuoka Cancer Center, 1007, Shimonagakubo, Nagaizumi-cho, Sunto-gun, Shizuoka, 411-8777 Japan; 2 Division of Rehabilitation Medicine, 1007, Shimonagakubo, Nagaizumi-cho, Sunto-gun, Shizuoka, 411-8777 Japan; 3 Division of Nutrition, 1007, Shimonagakubo, Nagaizumi-cho, Sunto-gun, Shizuoka, 411-8777 Japan; 4 Division of Physical Medicine and Rehabilitation, Shizuoka General Hospital, 4-27-1 Kita Ando Aoi-ku, Shizuoka City, 420-8527 Japan; 5 Department of Clinical Oncology, Osaka Medical Center for Cancer and Cardiovascular Diseases, 1-3-3 Nakamichi, Tosei-ku, Osaka, 537-8511 Japan; 6 Division of Respiratory Medicine, Gunma Prefectural Cancer Center, 617-1 Takabayashi-nishi-machi, Ohta-shi, Gunma, 373-8550 Japan; 7 Division of Cardiology, 1007, Shimonagakubo, Nagaizumi-cho, Sunto-gun, Shizuoka, 411-8777 Japan; 8 Division of Diagnostic Radiology, 1007, Shimonagakubo, Nagaizumi-cho, Sunto-gun, Shizuoka, 411-8777 Japan; 9 Division of Clinical Research Center, Cancer Center, 1007, Shimonagakubo, Nagaizumi-cho, Sunto-gun, Shizuoka, Shizuoka 411-8777 Japan; 10 Third Department of Internal Medicine, Wakayama Medical University, 811-1, Kimiidera, Wakayama-city, Japan

**Keywords:** Non–small cell lung cancer, Incremental shuttle walking distance, Hand-grip strength, Skeletal muscle mass, Sarcopenia, Cancer cachexia

## Abstract

**Background:**

Elderly patient with advanced cancer is one of the most vulnerable populations. Skeletal muscle depletion during chemotherapy may have substantial impact on their physical function. However, there is little information about a direct relationship between quantity of muscle and physical function. We sought to explore the quantitative association between skeletal muscle depletion, and muscle strength and walking capacity in elderly patients with advanced non–small cell lung cancer (NSCLC).

**Methods:**

Thirty patients aged ≥70 years with advanced NSCLC (stage III-IV) scheduled to initiate first-line chemotherapy were prospectively enrolled between January 2013 and November 2014. Lumbar skeletal muscle index (LSMI, cm^2^/m^2^), incremental shuttle walking distance (ISWD, m), and hand-grip strength (HGS, kg) were assessed at baseline, and 6 ± 2 weeks (T2) and 12 ± 4 weeks (T3) after study enrollment. Associations were analyzed using linear regression.

**Results:**

Altogether, 11 women and 19 men with a median age of 74 (range, 70–82) years were included in the study; 24 received cytotoxic chemotherapy and 6, gefitinib. Mean ± standard deviation of LSMI, ISWD and HGS were 41.2 ± 7.8 cm^2^/m^2^, 326.0 ± 127.9 m, and 29.3 ± 8.5 kg, respectively. LSMI and ISWD significantly declined from baseline to T2 and T3. HGS significantly declined from baseline to T2 and T3 only in men. Change in LSMI was significantly associated with change in HGS (β = 0.3 ± 0.1, *p* = 0.0127) and ISWD (β = 8.8 ± 2.4, *p* = 0.0005).

**Conclusions:**

Skeletal muscle depletion accompanied with physical functional decline started in the early phase of the chemotherapy in elderly patients with advanced NSCLC. Our results suggest that there may be a need for early supportive care in these patients to prevent functional decline during chemotherapy.

**Trial registration:**

Trial registration number: UMIN000009768

Name of registry: UMIN (University hospital Medical Information Network).

URL of registry: Date of registration: 14 January 2013.

Date of enrolment of the first participant to the trial: 23 January 2013.

## Background

The number of elderly people living with advanced lung cancer is increasing worldwide, owing to the aging population and advances in cancer treatment [1]. In Japan, 65% of lung cancer morbidity cases and 73% of annual lung cancer deaths were attributed to elderly individuals aged ≥70 years in 2012 [2]. Elderly patient with advanced cancer is one of the most vulnerable populations [3]. Patients with advanced non-small-cell lung cancer (NSCLC) frequently have cancer cachexia [4, 5] and skeletal muscle depletion [5, 6]. In addition, cancer treatment including radiotherapy [7], chemotherapy [8], and supportive care such as hospitalization [9] or the use of corticosteroids may cause muscle dysfunction [10]. Consequently, skeletal muscle depletion may cause physical dysfunction [11–14] and develop disability [15–17] before and during cancer treatment in NSCLC. Currently however, limited information exists on the quantitative association between loss of skeletal muscle mass and physical dysfunction in elderly patients with advanced NSCLC.

Accordingly, we sought to quantify impact of skeletal muscle mass depletion on muscle strength and walking capacity in elderly patients with advanced NSCLC receiving chemotherapy.

## Methods

### Patient selection

This prospective longitudinal observational study was performed at the Shizuoka cancer center, Japan, from January 2013 to January 2014. Shizuoka cancer center is a 615-bed prefectural hospital designated as an advanced treatment hospital by the Japanese Ministry of Health, Labor and Welfare. The eligibility criteria were as follows: (1) histologically and/or cytologically proven stage III or IV NSCLC including postoperative recurrence; (2) age ≥ 70 years, with planned first-line systemic chemotherapy; (3) no previous systemic chemotherapy or thoracic radiotherapy (adjuvant chemotherapy was not counted as a prior chemotherapy); (4) Eastern Cooperative Oncology Group performance status of 0–2; (5) ability to ambulate, read, and respond to questions without assistance; and (6) expected survival of >12 weeks. Patients were excluded if they had a severe psychiatric disorder, active infectious disease, unstable cardiac disease, or untreated symptomatic brain or bone metastases that prevented safe assessment.

All patients provided written informed consent. The study was approved by the institutional review board and registered on the clinical trials site of the University Hospital Medical Information Network Clinical Trials Registry in Japan (registration number: UMIN000009768).

### Patient enrollment and timing of data collection

The first patient was enrolled on January 23, 2013, and the last on November 7, 2013. The last physical assessment was performed on January 27, 2014. Lumbar skeletal muscle index (LSMI, cm^2^/m^2^), incremental shuttle walking distance (ISWD, m), and hand-grip strength (HGS, kg) were assessed at baseline (T1), and 6 ± 2 weeks (T2) and 12 ± 4 weeks (T3) after study enrollment. Baseline study assessments were performed by the attending physicians, physiotherapists, and national registered dietitians at the time between study entry and initiation of the first chemotherapy.

### Patient assessment

Body weight (kg) was measured to the nearest 0.1 kg and the body mass index (BMI; kg/m^2^) was subsequently calculated. The ISWD and HGS on the dominant side were measured by physiotherapists (T.O., T.O., Y.M., and T.I.). The incremental shuttle walking test was conducted according to the recent guideline [18] and original protocol described by Singh et al. [19]. The 10-m course was established in the corridor of our hospital. Walking speed was dictated by a timed signal played on a CD-recorder provided by the manufacturer (Japanese version, produced by the Graduate School of Biomedical Sciences, Nagasaki University, Japan, 2000). All patients were tested once under standardized conditions and were carefully observed during the test, so that they would not exceed their exercise limit. The instructor stayed alongside the course and provided no encouragement. The end of the test was determined by either (1) the patient, when he or she was too breathless to keep the required walking speed; (2) the instructor, if the patient could not complete a shuttle within the time allotted (ie, > 0.5 m away from the cone when the bleep sounded); or (3) attainment of 85% or higher of the predicted maximal heart rate derived from the formula [210 - (0.65 x age)]. The maximal walking distance was described as ISWD. Loss of 40 m was defined to be a clinically significant reduction in ISWD in this study [20]. HGS was measured using a grip strength dynamometer (GRIP-D, Takei Scientific Instruments Co., LTD, Niigata, Japan). Patient was in an upright position and held the dynamometer in one hand with the grip range adjusted so that the second joint of the forefinger was bent 90°. The instrument was then held down at the patient’s side without letting the arm touch the body, with the arm fully extended. Patient was then asked to exert full force with his or her hand for about 3 s to obtain the maximum kilogram-force, during which the instructor provided verbal encouragement. One trial was performed for each hand, and the result from the strongest hand was used for this analysis. Lumbar skeletal muscle mass was measured by analyzing electronically stored computed tomography images using SYNAPSE VINCENT version 3 (FUJIFILM Medical Systems, Japan). Conditions of CT image included contrast enhanced or unenhanced, 5-mm slice thickness. Two consecutive CT images at the third lumbar vertebra (L3) were chosen to measure the cross-sectional area of the skeletal muscle that was identified based on Hounsfield unit thresholds of −29 to +150. The sum of the cross-sectional areas (cm^2^) of the muscles in the L3 region was computed for each image. The mean value of 2 images was normalized for height in meters squared and reported as LSMI (cm^2^/m^2^) [21]. The disease stage was determined according to the TNM classification, and the best response to chemotherapy was evaluated according to the Response Evaluation Criteria in Solid Tumors.

### Diagnosis of muscle depletion and cancer cachexia

Skeletal muscle depletion was defined based on the cutoff point of the LSMI of 43 cm^2^/m^2^ for men with a BMI < 25.0, 53 cm^2^/m^2^ for men with a BMI ≥ 25.0, and 41 cm^2^/m^2^ for women [22].

Cancer cachexia was defined as unintentional weight loss >5% during the past 6 months or >2% in patients with a BMI <20 kg/m^2^, or the presence of muscle depletion according to the consensus criteria [23]. The patient’s weight 6 months before study entry was obtained by interviewing the patient and their family members at study entry.

### Statistical analysis

Chi-square or Fisher’s exact tests were used to compare categorical variables. Wilcoxon signed-rank test was used for the pairwise comparison of measurement changes between study visits, whereas the Wilcoxon rank-sum test was used for comparisons between 2 independent groups. For all analyses, *p*-values <0.05 were considered significant. All statistical analyses were performed using JMP version 12.0 for Windows (SAS Institute Inc., USA).

## Results

### Patients

Among 31 patients screened, 30 patients with a median age of 74 years (range, 70–82 years) were enrolled into this study; 11 patients (36.7%) were women (Table 1). Common comorbidities included chronic obstructive pulmonary disease, cardiovascular disease, and type 2 diabetes. There was a higher percentage never smokers among women than men (81.2 vs. 0%, *p* < 0.05).

**Table 1:** Baseline characteristics

Variables	All (*N* = 30)	Men (*N* = 19)	Women(*N* = 11)	Reference value (men/women)
Age, median (range)	74 (70–82)	74 (70–82)	76 (70–80)	
ECOG-PS, n (%)				
0	11 (36.7)	7 (36.8)	4 (36.4)	
1	18 (60.0)	12 (63.2)	6 (54.5)	
2	1 (3.3)	0 (0.0)	1 (9.1)	
Stage, n (%)				
IIIA or IIIB	1 (3.3)	1 (5.3)	0	
IV or postoperative reccurence	29 (96.7)	18 (94.7)	10 (100)	
Tumor Histology, n (%)				
Adenocarcinoma	21 (70.0)	13 (68.4)	8 (72.7)	
Other non-small-cell lung cancer	9 (30)	6 (31.6)	0	
Chemotherapeutic regimen, n (%)				
Cytotoxic regimen	24 (80.0)	17 (89.5)	7 (63.6)	
Targeted regimen	6 (20.0)	2 (10.5)	4 (36.4)	
Never smoke, n (%)	9 (30.0)	0*	9 (81.2)	
Comorbidities, n (%)				
Chronic obstructive pulmonary disease	10 (33.3)	7 (36.8)	3 (27.3)	
Type 2 diabetes	6 (20.0)	5 (26.3)	1 (9.1)	
Cerebrovascular disease	4 (13.3)	4 (21.1)	0	
Cardiovascular disease	1 (3.3)	0	1 (9.1)	
Body composition				
Body-mass index (kg/m^2^)	21.1 ± 3.4	21.6 ± 3.5	20.2 ± 3.1	
Lumbar skeletal muscle index (cm^2^/m^2^)	41.2 ± 7.8	44.5 ± 7.6*	35.4 ± 4.1	
Skeletal muscle depletion^a^, n (%)	20 (66.7)	10 (52.6)*	10 (90.9)	17.2/ 19.9 [26]
Cancer cachexia^b^, n (%)	19 (63.3)	11 (57.9)	8 (72.7)	
Physical function				
Hand grip strength (dominant side, kg)	29.3 ± 8.5	33.9 ± 7.1*	21.7 ± 4.1	32/ 20 [24]
Shuttle walk distance (m)	326.0 ± 127.9	338.4 ± 143.0	304.5 ± 99.2	360–400 [25]

*Significant gender difference (*P* < 0.05) tested by Chi-square test, Fisher exact test, or Wilcoxon test. ^a^skeletal muscle mass depletion was defined as lumbar skeletal muscle mass index of <43.0 cm^2^/m^2^ for men with a BMI <25.0 kg/m2, <53.0 cm^2^/m^2^ for men with a BMI ≥25.0, and <41.0 cm^2^/m^2^ in women ^b^Diagnosis was based on the international consensus criteria for cancer cachexia. ECOG-PS: Eastern cooperative oncology group performance status

### Cancer treatment during the study period

All patients received first-line chemotherapy within 1 week after the baseline assessment. All patients initially received a standard dose of chemotherapy with a standard schedule. Ten patients received single-agent chemotherapy, including docetaxel (60 mg/m^2^, every 3 weeks, *n* = 8) and vinorelbine (25 mg/m^2^, day 1 and 8 every 3 weeks, *n* = 2), until disease progression or unacceptable toxicity. Median treatment cycle (range) was 5 (2–12) cycles. Two patients required dose reduction due to febrile neutropenia and moderate nausea. One patient discontinued chemotherapy after 2 cycles of docetaxel due to performance status deterioration and bacterial pneumonia. Fourteen patients received platinum-based chemotherapy, including 7 patients who received carboplatin (target area under the curve of 6, every 3 weeks) + paclitaxel (200 mg/m^2^, every 3 weeks), 5 who received cisplatin (75 mg/m^2^, every 3 weeks) + pemetrexed (500 mg/m^2^, every 3 weeks), 1 who received cisplatin (80 mg/m^2^, every 3 weeks) + gemcitabine (1000 mg/m^2^, day1 and 8 every 3 weeks), and 1 patient cisplatin (80 mg/m^2^, every 3 weeks) + vinorelbine (25 mg/m^2^, day1 and 8 in every 3 weeks). Treatment was planned for 4 to 6 cycles unless there was evidence of unacceptable toxicity or disease progression. Median cycle (range) was 4 (2–6) cycles. Two patients required dose reduction due to elevated serum creatinine level and severe weight loss. One patient changed his regimen after 2 cycles of carboplatin + paclitaxel due to a severe allergic reaction. Six patients with epidermal growth factor receptor gene mutations received gefitinib (250 mg once daily). The median treatment period was 10.2 months. One patient required a dose reduction due to moderate liver dysfunction. None of the patients discontinued treatment due to adverse events. An objective tumor response was seen in 12 patients (40.0%).

### Evaluable patient data

**Fig. 1:**
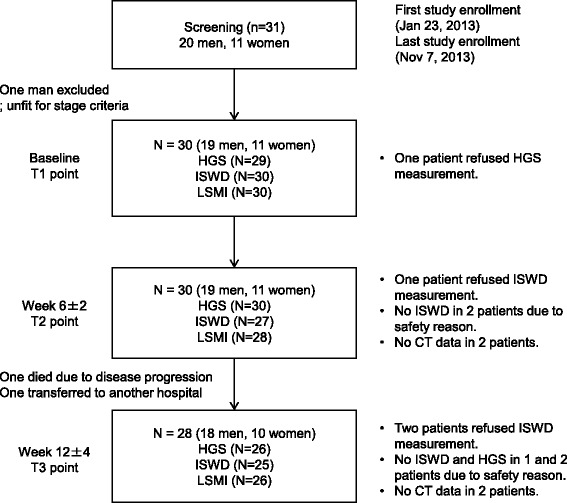
Flow diagram. The number of patients and evaluable data at the T1 (baseline), T2 (6 ± 2 weeks), and T3 (12 ± 2 weeks) point is shown. The number of evaluable data for each variable is described in the box. The reasons for a missing value are described in the right side of each box. HGS, hand-grip strength; ISWD, incremental shuttle walking distance; LSMI, lumbar skeletal muscle index

Patient flow and the number of evaluable data at each time point are summarized in the flow diagram (Fig. 1). Among 30 patients enrolled, 30 and 28 patients were alive and eligible for evaluation at T2 and T3, respectively. One man died from disease progression and one woman was transferred to another hospital until T3 point. At baseline, the HGS test was refused by a patient. At T2 point, the shuttle walking test was refused by one patient and abandoned by the physiotherapist in 2 patients for safety reason; and computed tomography data in 2 patients were not obtained during the designed period. At T3 point, the shuttle walking test was refused by 2 patients and abandoned by the physiotherapist in one patient for safety reason; the HGS test was abandoned by the physiotherapist in 2 patients for safety reason; and computed tomography data in 2 patients were not obtained during the designed period.

### Body mass, muscle mass, and physical function at baseline

At baseline, mean ± standard deviation of BMI was 21.5 ± 3.4 kg/m^2^ in men and 20.1 ± 3.1 kg/m^2^ in women (Table 1). Mean LSMI was 44.5 ± 7.6 cm^2^/m^2^ in men and 35.4 ± 4.1 cm^2^/m^2^ in women with a significant difference between the sexes (*P* < 0.05). Skeletal muscle depletion was diagnosed in 20 (66.7%) patients and higher proportion of women were diagnosed with skeletal muscle depletion than men (90.9 vs. 52.6%, *p* < 0.05). Cancer cachexia was diagnosed in 19 (63.3%) patients. In regard to the physical function, mean HGS was 33.8 ± 7.0 kg in men and 21.7 ± 4.0 kg in women with a significant difference between the sexes (*P* < 0.05). These values were almost comparable to the reference value in the Japanese community-dwelling elderly population [24] (shown in Table 1). Mean ISWD was 338.4 ± 142.9 in men and 304.5 ± 99.2 in women without gender difference (*P* = 0.54). The values were relatively low in comparison with the reference values in the Japanese community-dwelling elderly population [25].

### Longitudinal changes in muscle mass and physical function

**Fig. 2:**
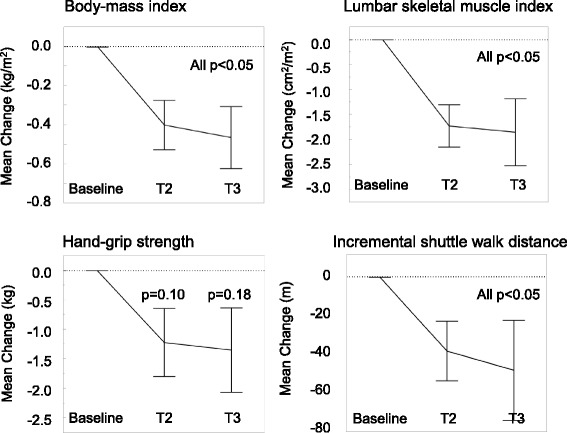
Longitudinal changes in body-mass, muscle mass, and physical function. Mean changes ± standard error of physical parameters from baseline value is shown. *P*-value of Wilcoxon signed-rank test was shown

Statistically significant reductions between baseline values, and T2 and T3, were seen for weight, BMI, LSMI, and ISWD. A clinically significant reduction in ISWD was also seen in 11 patients (40.7%) at T2 and in 13 patients (52.0%) at T3. No statistically significant reductions were observed between T2 and T3 for weight, BMI, LSMI, HGS, and ISWD (Table 2 and Fig. 2). Men had a significant reduction in HGS at T2 (*p* < 0.05) and T3 (*p* < 0.05), whereas women had no reduction in either time point (*p* = 0.45 and *p* = 0.78, respectively).

**Table 2:** Longitudinal changes in physical parameters

Variables	Mean difference from baseline (±SE)		Mean difference between T2 and T3 (±SE)
	6 ± 2wks	12 ± 4wks	
N	30	28	25
Body weight (kg)	−0.9 ± 0.4*	−1.1 ± 0.6*	−0.2 ± 0.4
Body-mass index (kg/m^2^)	−0.3 ± 0.1*	−0.4 ± 0.1*	−0.1 ± 0.1
L3 muscle index (cm^2^/m^2^)	−1.8 ± 0.4*	−1.8 ± 0.7*	−0.1 ± 0.4
Hand grip strength (non-dominant, kg)	−0.7 ± 0.6	−0.7 ± 0.6	−0.5 ± 0.3
Shuttle walk distance (m)	−40.0 ± 12.6*	−46.4 ± 15.8*	−10.8 ± 11.3
Clinically significant decline^b^, n (%)	11 (40.7)	13 (52.0)	5 (20.0)

*p < 0.05 in Wilcoxon signed-rank test compared with baseline value

^b^Clinically significant decline is defined as losing ≥40 m of shuttle walk distance from baseline

### Association between changes in skeletal muscle mass and physical function

**Fig. 3:**
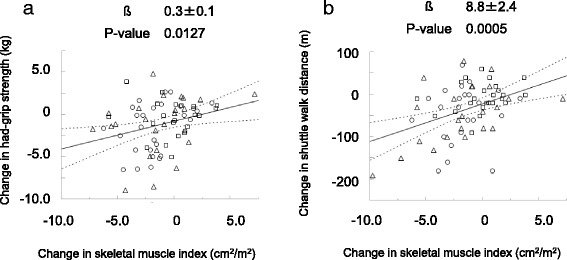
Association between changes in skeletal muscle mass and physical function. The association between change in muscle mass, and hang-grip strength (**a**) and shuttle walking distance (**b**) at all time points are plotted. Dotted line indicates the 95% confidence interval. Circle, triangle, and square mark represents change at T2 from baseline, T3 from baseline, and T3 from T2, respectively

There was a statistically significant linear association between changes in LSMI and HGS (β = 0.3 ± 0.1, *p* = 0.0127, Fig. 3a). There was also a positive linear association between LSMI and changes in HGS (β = 8.8 ± 2.4, *p* = 0.0005, Fig. 3b).

### Subset analysis for changes in skeletal muscle mass at T2 point

**Fig. 4:**
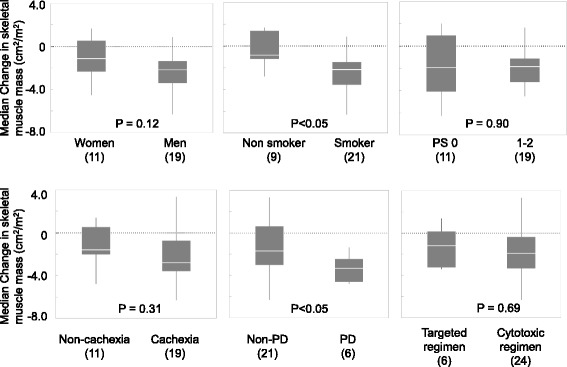
Subset analysis for change in skeletal muscle mass at T2 point. Median change of skeletal muscle mass at T2 point in each subset was shown. The number of patients in each subset is indicated in parenthesis. White line indicates the median. The top and bottom of each box represent the upper and lower quartiles of the values for the sample. Bars extend above and below each box to the maximal and minimal values in the sample. *P*-value of Wilcoxon rank-sum test was shown. PS, Eastern Cooperative Oncology Group performance status; PD, progressive disease assessed by the Response Evaluation Criteria in Solid Tumors at T2 point

In subset analysis in LSMI at T2 point, depletion in LSMI was observed in most of the subsets classified by gender, smoking status, performance status, presence of cancer cachexia, response to chemotherapy, and treatment regimens (Fig. 4). Smokers had a larger reduction in LSMI than never-smokers (*P* < 0.05). Similarly, patient with tumor progression at T2 had larger reduction in LSMI than patients without tumor progression (*P* < 0.05). There was no statistical association between treatment modification (dose reduction or discontinuation) and reduction in LSMI.

## Discussion

To our knowledge, this is the first prospective study to show longitudinal changes in skeletal muscle mass associated with physical function in elderly patients with advanced NSCLC receiving chemotherapy. First, we showed that majority of this patient population had skeletal muscle depletion, cancer cachexia, and decreased walking capacity at baseline. Second, we found that they rapidly lost their body mass, skeletal muscle mass, muscle strength, and walking capacity in the early course of systemic chemotherapy. Third, we found positive associations between changes in skeletal muscle mass and muscle strength or walking capacity.

Dewys WD et al. [4] reported that two-thirds of incurable chemotherapy-naïve patients with NSCLC experienced weight loss, especially in those with advanced disease. In our previous research, we reported that 45.6% of chemotherapy-naïve patients with advanced NSCLC had cancer cachexia at baseline [5]. The incidence of cancer cachexia in the present study (63.3%) was somewhat higher. The possible reasons for this difference is that this study only included elderly patients (median age, 74 years vs. 66 years in our previous study) and more patients with metastatic disease (97% vs. 88%). High incidences of sarcopenia have been reported in patients with advanced lung cancer [5, 6]. Consistently, our patients had relatively high incidence of skeletal muscle depletion (52.6% in men and 90.9% in women), compared with those of community-dwelling Japanese elderly population (17.2% in men and 19.9% in women [26]). In addition, patients with advanced lung cancer have been reported to have poorer physical function, compared with community-dwelling elderly in regards to muscle strength and endurance performance measured by the 6-min walking test [12, 13, 27]. In this study, the baseline values of the incremental shuttle walking distance tended to be lower, compared with the reference values of community-dwelling elderly populations [25].

Weight loss during cancer treatment is commonly observed in patients with lung cancer receiving chemotherapy [5, 28] or thoracic radiotherapy [7], and is accompanied by a marked decrease in skeletal muscle mass [5, 8]. Similarly, our patients had a significant decrease in body mass and skeletal muscle mass during the first 6–12 weeks of cancer treatment. Stene GB et al. [8] reported that patients with disease progression following chemotherapy tended to have a larger reduction in skeletal muscle mass, compared with patients with disease control following chemotherapy. Our data also showed that patients with tumor progression had greater muscle loss in the subset analysis.

Change in walking capacity during chemotherapy or radiotherapy has rarely been described in patients with advanced lung cancer. Kasymjanova et al. reported that 6-min walking distance significantly declined after 2 cycles of systemic chemotherapy with or without radiotherapy in patients with advanced NSCLC. They reported a 30% dropout rate during follow-up evaluation mainly due to patients being too ill to complete the test, or because they had died [13]. However, 29% of patients who completed the study had a clinically significant (>54 m) decline in walking distance. In our study, 3 patients (10.0%) at T2 and 5 patients (16.6%) at T3 dropped out of follow-up assessment of ISWD mainly due to disease progression. Among those who completed the study, 40.7% patients at T2 and 52.0% at T3 showed clinically significant reduction in ISWD (≥40 m). Older age and worse disease burden may elevate the proportion of deterioration in walking capacity.

Our study has several limitations. First, this was a small study that included only Japanese patients treated at a single institution. Second, our study population was heterogeneous in regard to the treatment regimens received. This may have affected the physical or nutritional changes analyses. Patients who receive platinum-based chemotherapy and are treated with a steroid antiemetic in hospital may be much more vulnerable to treatment-related muscle dysfunction, compared with patients receiving oral targeted treatment (e.g. gefitinib) on an outpatient basis. However, this had little impact on the comparison of endpoints in this study.

There is only a limited evidence about an early nutritional and exercise intervention for the patients with advanced cancer who are receiving chemotherapy [29, 30]. One of the reasons for this is a lack of information about the longitudinal changes in body composition and its impact on physical function during chemotherapy for specific cancer types. Recently, Kaasa S et al. [31] reported the results of a randomized phase II study comparing a multimodal intervention (exercise, nutritional intervention, and anti-inflammatories) versus standard cancer care in patients with advanced NSCLC and pancreatic cancer (Pre-MENAC study, Clinical Trials Registry No. NCT01419145). They showed that the intervention was feasible and was associated with statistically significant weight gain. However, there was no significant improvement in muscle mass and physical activity. The MENAC study, a phase III randomized, open-label trial of this multimodal intervention plus standard care vs. standard care alone to prevent cachexia in advanced cancer patients undergoing chemotherapy, is now underway (Clinical Trials Registry No. NCT02330926). Based on the results of our study, we further narrow the study population to the elderly patients and are now conducting a prospective multicenter study of early exercise and nutritional intervention for advanced NSCLC and pancreatic cancer in Japan (Clinical Trials Registry No.UMIN000023207).

## Conclusion

Skeletal muscle depletion accompanied with physical functional decline started in the early phase of the chemotherapy in elderly patients with advanced NSCLC. Our results suggest that there may be a need for early supportive care in these patients to prevent functional decline during chemotherapy. Further randomized control study is needed to determine whether early exercise and nutritional intervention may be useful to prevent muscle depletion and functional decline in this population.

## Abbreviations

BMI: Body mass index
HGS: Hand grip strength
ISWD: Incremental shuttle walking distance
LSMI: Lumbar skeletal muscle index
NSCLC: Non–small cell lung cancer
